# A Novel Minimally Invasive Robotic-Assisted Surgery Technique for Treatment of Median Arcuate Ligament Syndrome: A Case Report

**DOI:** 10.7759/cureus.66933

**Published:** 2024-08-15

**Authors:** Natalia M Barron-Cervantes, Alejandro Martinez-Esteban, Erik F Gardner-Hilbert, Eduardo Villegas-Tovar, Regina Faes-Petersen, Alejandro D. G. Gidi

**Affiliations:** 1 General and Gastrointestinal Surgery Service, Fundación Clínica Médica Sur, Mexico City, MEX; 2 General and Gastrointestinal Surgery Service, Fundación Clínica Medica Sur, Mexico City, MEX; 3 General and Gastrointestinal Surgery, Angeles Health System, Mexico City, MEX

**Keywords:** chronic abdominal pain, dunbar syndrome, celiac artery compression, abdominal pain, mals, median arcuate ligament syndrome

## Abstract

Median arcuate ligament syndrome (MALS), also known as Dunbar syndrome, is a rare but significant cause of chronic abdominal pain resulting from the extrinsic compression of the celiac trunk. This condition typically manifests with symptoms such as postprandial pain, nausea, vomiting, and weight loss, often leading to a diagnostic challenge due to its mimicry of other abdominal disorders. Diagnosis is based on exclusion and requires a high index of suspicion combined with precise imaging findings. This case report presents a 44-year-old female patient presenting with chronic abdominal pain, diaphoresis, and nausea, underscoring the complexity and diagnostic challenge of MALS, highlighting the significance of early intervention to mitigate morbidity and novelty treatment utilizing robotic-assisted surgical techniques. This report aims to contribute to the understanding of clinical presentations, diagnostic challenges, and treatment modalities of Dunbar syndrome, especially the option of minimally invasive robotic-assisted surgery for the treatment of this condition.

## Introduction

Dunbar syndrome, also known as median arcuate ligament syndrome (MALS), is a rare condition that involves the extrinsic compression of the celiac trunk by a low-riding fibrous attachment of the median arcuate ligament and the diaphragmatic crura. MALS diagnosis is based on exclusion in patients presenting with chronic abdominal pain, which includes vaguely comprising postprandial symptoms, nausea, vomiting, and weight loss [1]. As Dunbar syndrome mimics many possible abdominal disorders, its diagnosis is complex and usually underdiagnosed. Throughout this case presentation, the complexity of the diagnostic approach will be exposed, as well as the importance of the treatment based on the decompression of the celiac artery in order to establish adequate proper blood flow and pain management by neurolysis [2]. Another important thing to emphasize is that Dunbar syndrome presents a difficult diagnosis that requires a high grade of suspicion mixed with accurate supportive imaging findings [3].

During this case study, the significance of early treatment is presented. MALS management is based on surgical treatment. It can be an open decompression by the dissection of the diaphragmatic crura from the celiac axis or a laparoscopic approach. Other options include the removal or ablation of the ganglion and celiac artery revascularization in order to approach the neuropathic pain. The role of interventional radiology (IV) has been emerging in the last decades, principally the use of percutaneous transluminal angioplasty (PTA) as an adjuvant therapy prior to surgery [2]. We present the case of a 44-year-old female patient who presented to the emergency department (ED) with chronic abdominal pain accompanied by diaphoresis and nausea. This case was presented at a first-level private surgical center in Mexico City. This case is handed out in order to further expand the knowledge about the clinical presentation of the Dunbar syndrome as well as highlight the importance of an early approach to improve morbidity in these cases.

## Case presentation

A previously healthy 44-year-old female presented to the emergency department (ED) with an episode of epigastric pain (9/10), with no radiation and without any identified triggers or causes that may be associated with improvement or worsening of the symptoms. After the initial evaluation in the ED was performed, the patient was questioned about other symptoms presented in the past to which she reported that she had had similar symptoms for the last two years on approximately seven occasions, all of them self-limiting, which is why she had not sought medical attention. As accompanying symptoms, she presented episodes of diaphoresis and nausea predominantly postprandial since the beginning of the first clinical presentation. Due to the persistence of symptoms and worsening pain in the current episode, she decided to go to the ED. During the physical examination (PE) in the ED the patient was alert and oriented, skin and mucous membranes with apparently good hydration and coloration. There were no signs suggestive of peritonitis. The patient's family history includes hypertension, and her mother had an unspecified type of epileptic syndrome and autoimmune hypothyroidism. Within her past medical history, it is important to mention that she has the diagnosis of endometriosis without current treatment and non-autoimmune primary hypothyroidism managed with oral levothyroxine. Likewise, she has a surgical history of a right oophorectomy three years prior to the current condition secondary to an ovarian torsion and a cesarean section performed in 2014.

The decision to hospitalize the patient in order to continue the diagnostic approach was made. Due to the high suspicion of cholecystitis, the patient was kept with nothing per oral (NPO) and with intravenous (IV) Ringer’s lactate solution. Blood laboratories and magnetic resonance imaging (MRI) of the upper abdomen with IV gadolinium contrast were requested. Laboratories were remarkable for hyperglycemia and mildly elevated hepatic enzymes (Table 1).

**Table 1:** Laboratory exams. CBC, relevant data from blood chemistry (BC), hepatic profile, and coagulation times. INR, international normalized ratio; BUN, blood urea nitrogen; PT, prothrombin time; ALT, alanine aminotransferase; AST, aspartate aminotransferase; GGT, gamma-glutamyl transpeptidase; ALP, alkaline phosphatase; LDH, lactate dehydrogenase; CBC, complete blood count; BC, blood chemistry

Parameter	Value	Reference values
Hemoglobin	13.1 g/dL	12 - 18 g/dL
Hematocrit	38.2%	36 - 50%
Platelets	244 x 10^3^/uL	150 - 450 x 10^3^/uL
Leukocyte count	6.8 x 10^3^/uL	4.5 - 11 x 10^3^/uL
Absolute neutrophils	5 x 10^3^/uL	2.5 - 7 x 10^3^/uL
Serum sodium	141 mmol/L	135 - 145 mmol/L
Serum potassium	4.4 mmol/L	3.6 - 5.2 mmol/L
Serum glucose	138 mg/dL	72-99 mg/dL
Serum creatinine	0.55 mg/dL	0.31-1.00 mg/dL
BUN	12.9 mg/dL	7-22 mg/dL
PT	9.3 seconds	11 - 13.5 seconds
INR	1.01	<1.1
Total bilirubin	0.68 mg/dL	1 - 1.2 mg/dL
Indirect bilirubin	0.39 mg/dL	0.2 - 1.2 mg/dL
ALT	42 U/L	5 - 40 U/L
AST	77 U/L	4 - 36 U/L
GGT	48.5 U/L	0 - 30 U/L
ALP	75.8 U/L	44 - 147 U/L
LDH	175 U/L	140 - 280 U/L

**Figure 1:**
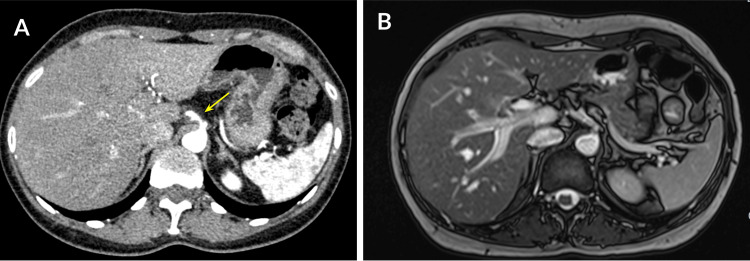
Upper abdominal IV contrast CT and an MRI with IV contrast with gadolinium. A) Arterial phase of IV contrast CT showing a filling defect in the dorsal region of the celiac trunk (yellow arrow). B) Contrast-enhanced T2 MRI demonstrating stenosis that caused a reduction in arterial blood flow of up to 50% in a 5.6 mm segment with a deflection angle of the celiac trunk of 79°. MRI, magnetic resonance imaging; IV, intravenous; CT, computed tomography

The MRI reported a left parasagittal vascular stenotic formation observed in the dorsal region of the celiac trunk. This vascular stenosis was detected to cause a reduction in arterial blood flow of up to 50% in a 5.6 mm segment with a deflection angle of the celiac trunk of 79° (Figure 1).

**Figure 2:**
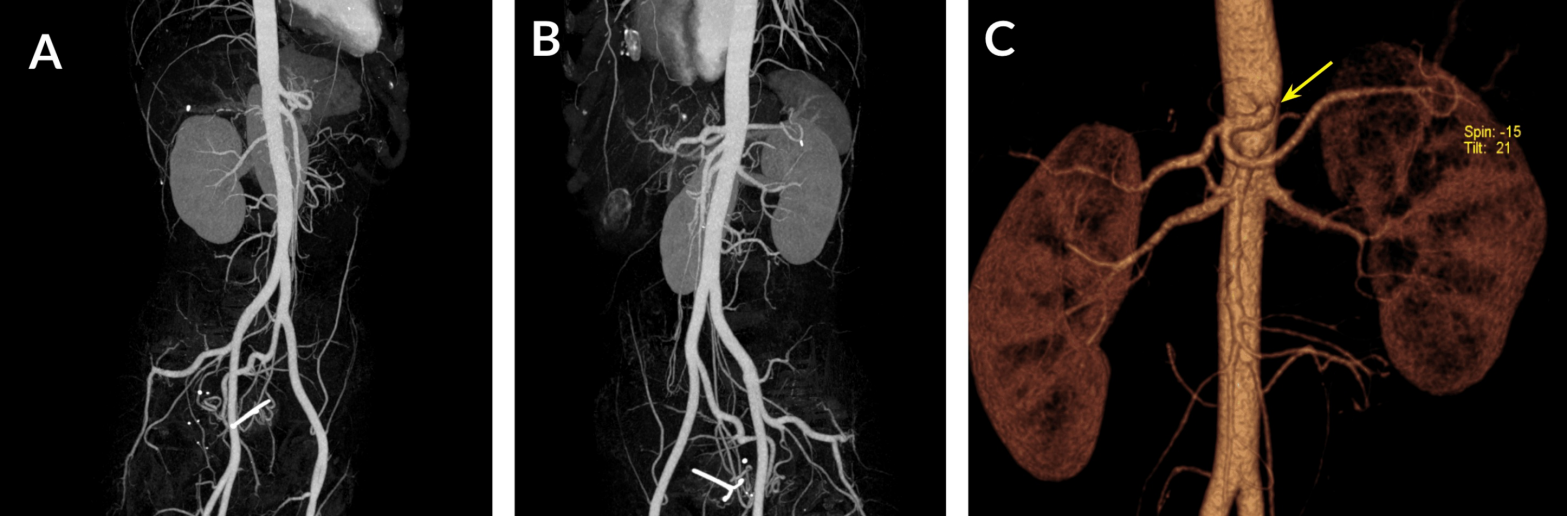
Three-dimensional reconstruction of the upper abdominal contrasted MRI. Decrease in the lumen of the caliber of the celiac artery secondary to extrinsic compression. A) Right lateral view. B) Left lateral view. C) Anterior view. Yellow arrow: filling defect in the dorsal region of the celiac trunk caused by extrinsic compression by the middle arcuate ligament. MRI, magnetic resonance imaging

**Figure 3:**
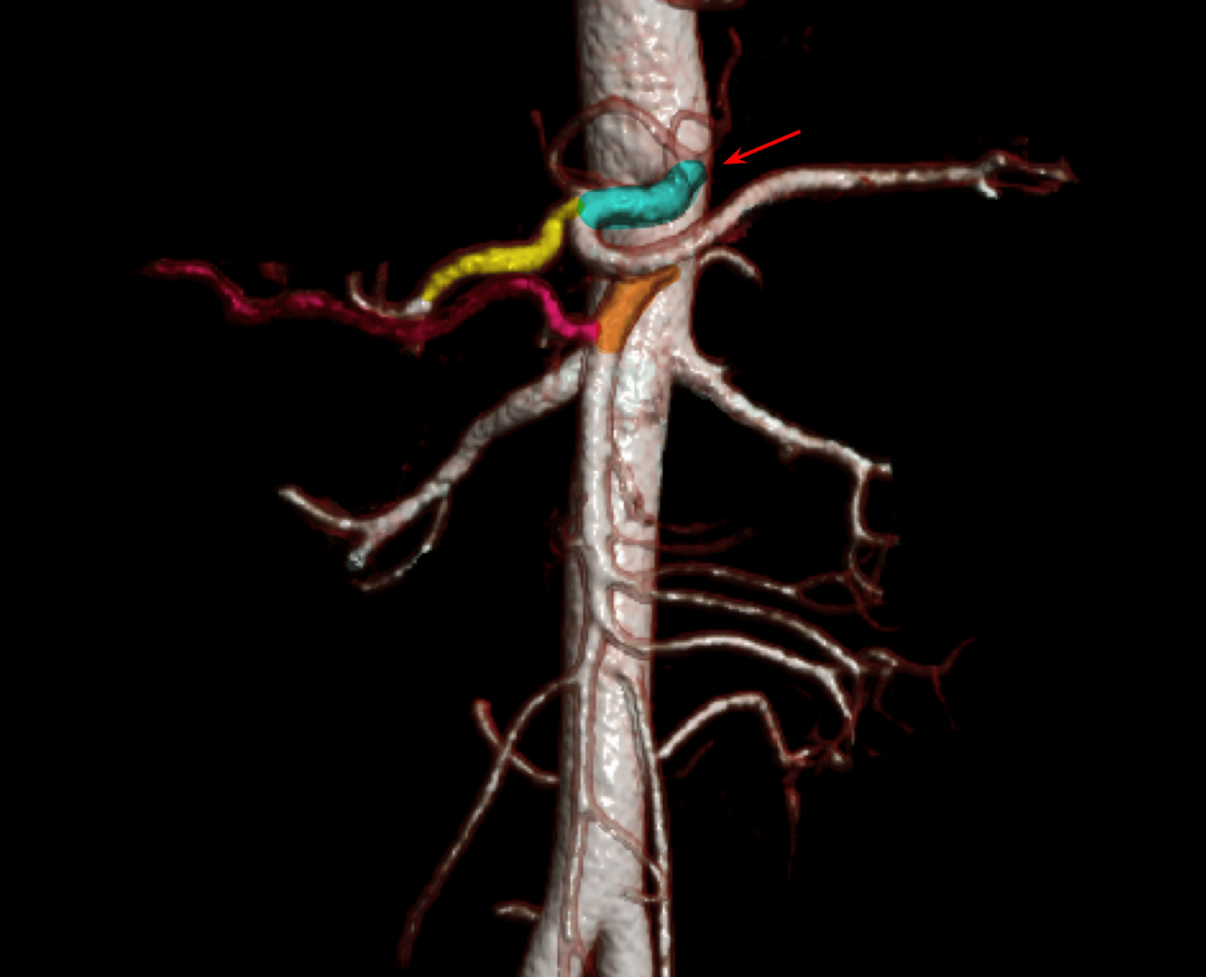
Right hepatic artery replacement. Three-dimensional reconstruction of the upper abdominal contrasted MRI shows that the right hepatic artery (pink artery) is a replacement for the SMA (orange artery). Filling defect in the dorsal region of the celiac trunk caused by extrinsic compression by the middle arcuate ligament (red arrow). The rest of the anatomy shows no abnormalities, with the celiac trunk (blue artery) giving the branch of the common hepatic artery (yellow artery) and its subsequent branches. SMA, superior mesenteric artery

With the clinical presentation and the images shown in the MRI, the diagnosis of MALS was established. In order to prepare the patient for surgical resolution, a three-dimensional reconstruction was made from the contrasted MRI images. Within this reconstruction, a decrease in the lumen of the caliber of the celiac artery secondary to extrinsic compression was observed, with no evidence of calcifications or atherosclerosis in the same vessel (Figure 2). As an incidental finding during this three-dimensional reconstruction scan, it was identified that the right hepatic artery is a replacement for the superior mesenteric artery (SMA) (Figure 3).

**Figure 4:**
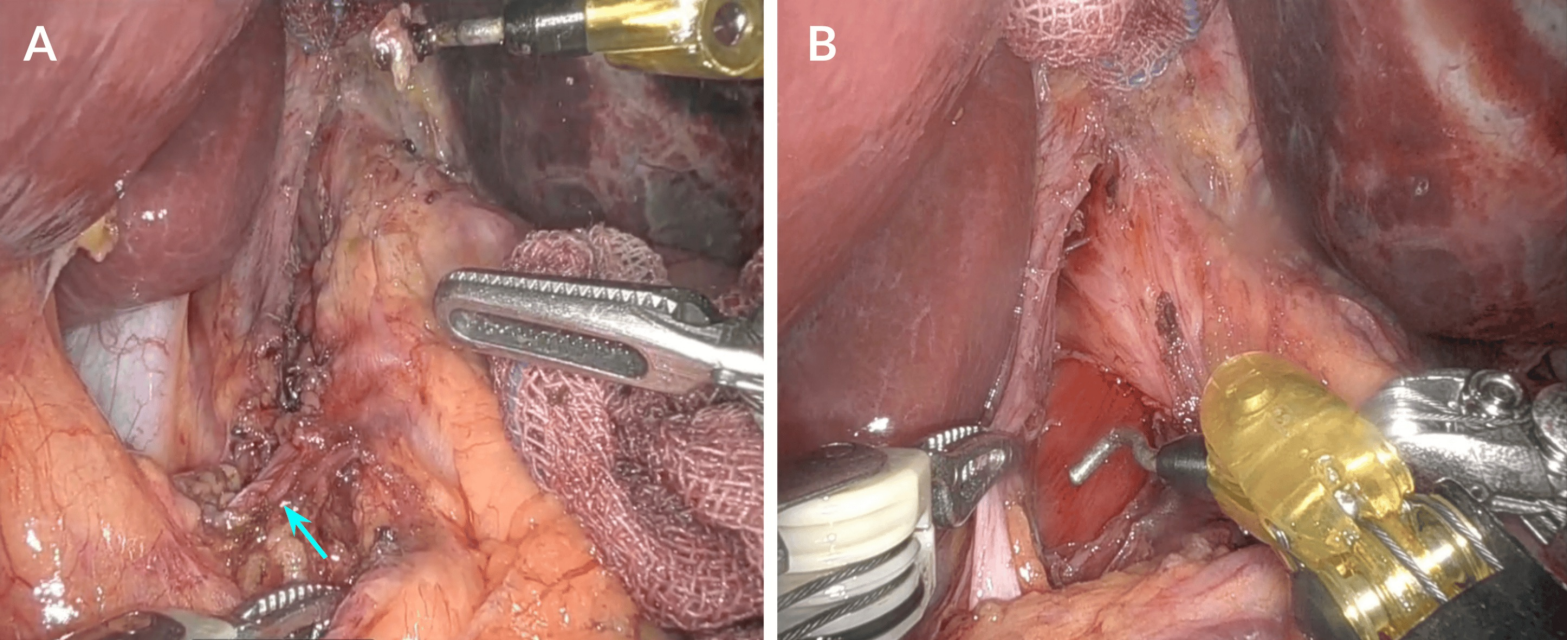
Visualization and exposure of the celiac artery during laparoscopic surgery. A) The stomach and duodenum were dissected in order to expose the celiac artery (blue arrow) as it passes through the diaphragmatic crura and under the median arcuate ligament. B) Dissection of the hepatogastric ligament and retroesophageal dissection in order to expose the abdominal aorta and subsequently the birth of the celiac trunk.

The patient was scheduled for laparoscopic decompression of the celiac artery. During the surgery, the peritoneal cavity was inspected to confirm the diagnosis and rule out any other abdominal pathology. Then, the stomach and duodenum were visualized in order to identify the celiac artery as it passes through the diaphragmatic crura and under the median arcuate ligament. After identifying those structures, the hepatogastric ligament and retroesophageal space were dissected in order to expose the abdominal aorta and subsequently the birth of the celiac trunk (Figure 4).

**Figure 5:**
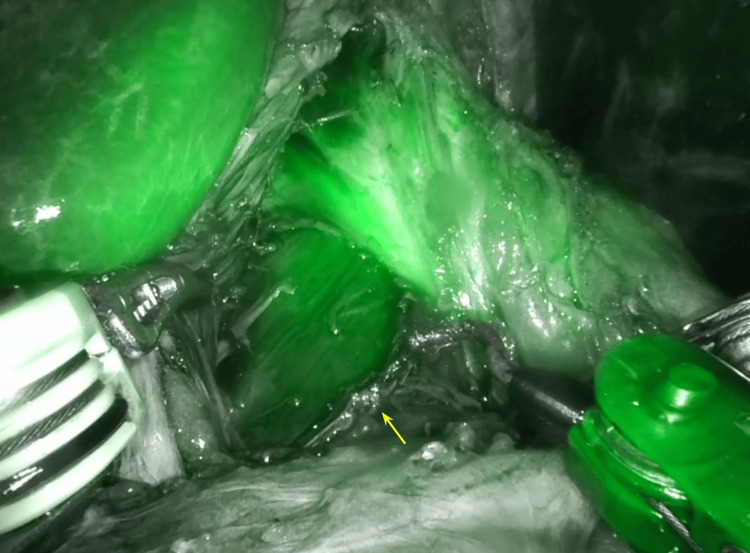
Recognition of the abdominal vasculature from the use of indocyanine green. The administration of indocyanine green was performed in order to be able to correctly observe the vasculature at the aortic level and to positively identify the celiac trunk (yellow arrow).

**Figure 6:**
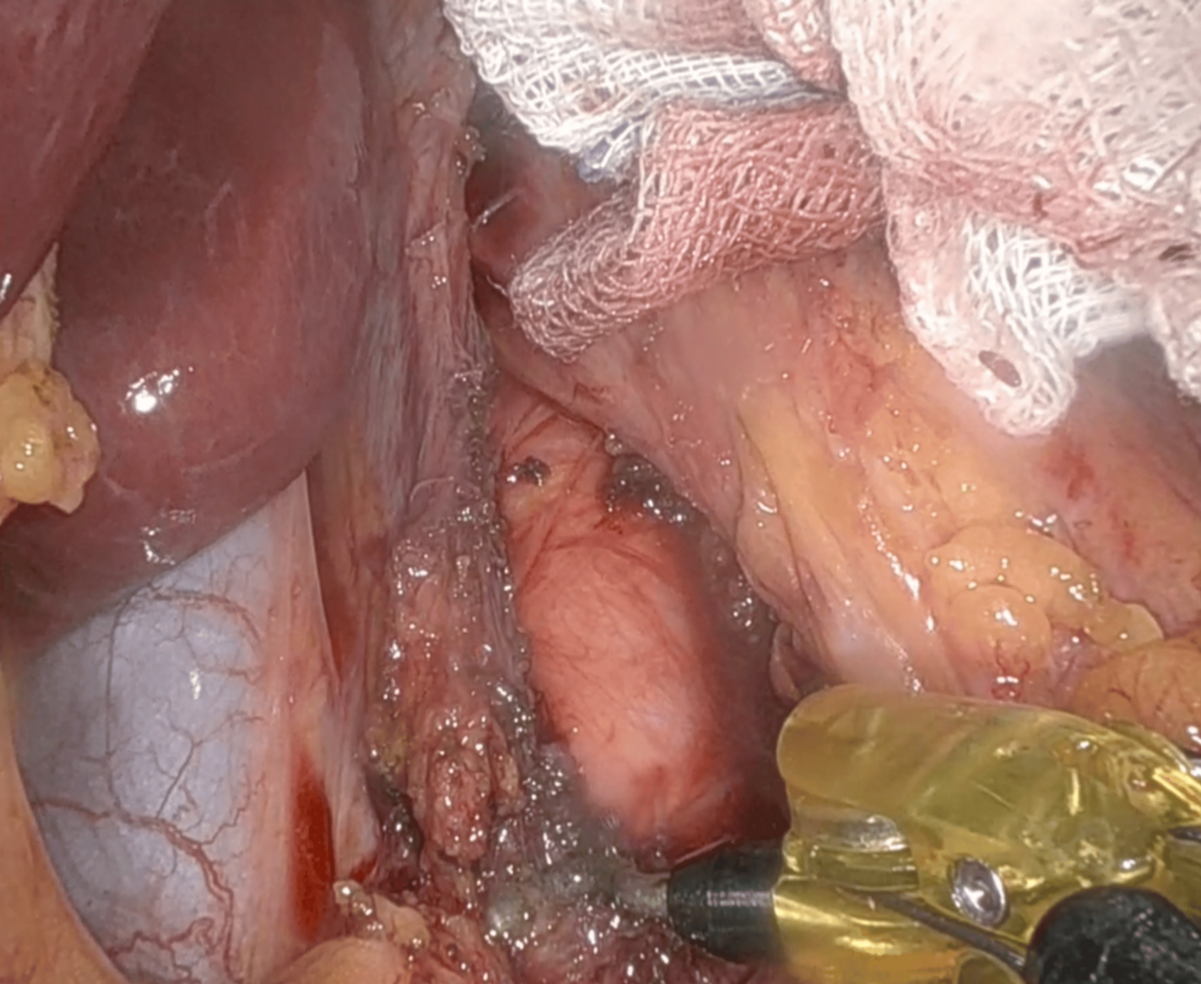
Dissection of the median arcuate ligament. Dissection of the upper portion of the median arcuate ligament.

The subsequent phase in the surgical procedure involved the identification of the abdominal vasculature utilizing indocyanine green (Figure 5). After correctly recognizing the celiac artery, the median arcuate ligament was identified and started to be dissected. Careful dissection was carried out to separate the ligament from the celiac artery and surrounding tissues (Figure 6).

**Figure 7:**
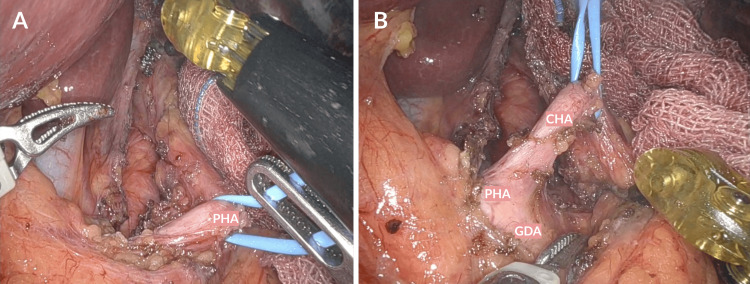
Dissection of the branches of the celiac trunk. A) Identification and vascular control of the proper hepatic artery, marked as PHA. B) Identification and vascular control of the PHA, the GDA, and the CHA. PHA, proper hepatic artery; GDA, gastroduodenal artery; CHA, common hepatic artery

After the upper portion of the median arcuate ligament was free, the vascular dissection was performed. For this part of the dissection first, the celiac trunk was identified and separated from the surrounding structures and then its main branches were identified and dissected (Figure 7).

**Figure 8:**
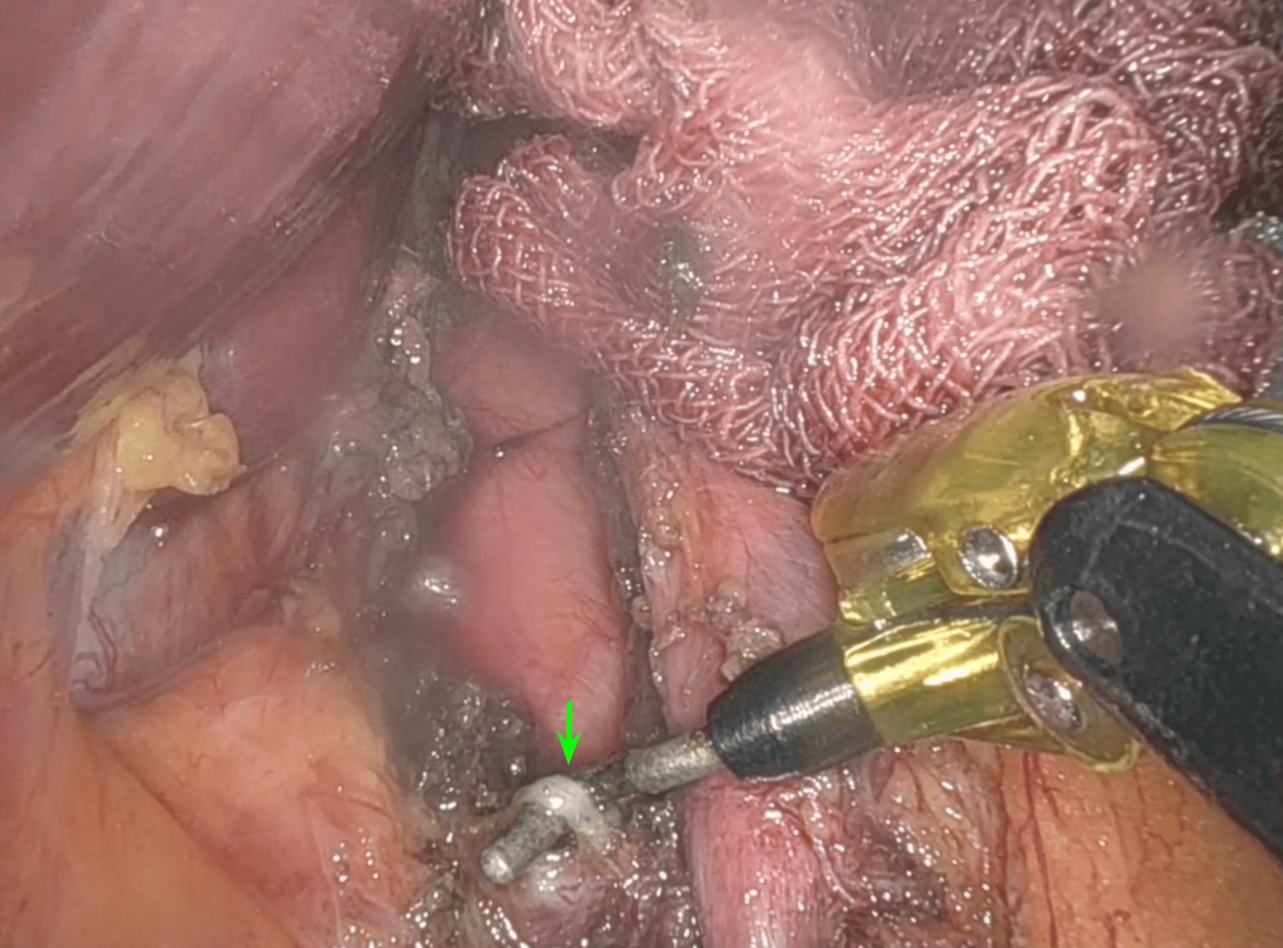
Decompression of the median arcuate ligament. The dissection of the median arcuate ligament at the supraceliac level (green arrow) is completed with periaortic dissection.

Finally, the median arcuate ligament is completely dissected and released (Figure 8). After that, the celiac artery is inspected to ensure that it is freely mobile and not under tension; this step aims to restore normal blood flow to the celiac artery and alleviate symptoms caused by compression. The final surgical procedure was established as a celiac trunk dissection with decompression of the median arcuate ligament.

Following surgery, the patient's diet was advanced incrementally without manifestation of pain or associated complications. Subsequent to the procedure, all symptoms abated entirely. The patient experienced an uneventful hospital course and was discharged 48 hours postoperatively, devoid of any adverse events. At the three-month follow-up appointment, the patient reported complete weight restoration, heightened appetite, and absence of symptoms resembling those observed over the preceding two years.

## Discussion

MALS is a syndrome characterized by the compression of the celiac trunk by a low median arcuate ligament and diaphragmatic crura. The first description of this condition was made by Dr. Lipshutz in 1917, who performed cadaveric dissections and showed the overlapping of the celiac artery leading to the compression of the celiac trunk, presented in people who englobed the same group of vague symptoms [4]. Later on, Dr. Harjola reported a case report of a 57-year-old patient with postprandial epigastric pain and after the decompression of the celiac artery, symptoms were completely relieved [5]. The celiac axis usually arises from the abdominal aorta at the vertebral levels of T11 to T12; however, many anatomic variations have been described. The median arcuate ligament is a band of fibrous tissue that connects the diaphragmatic crura anteriorly and surrounds the abdominal aorta at the level of the aortic hiatus. There are two anatomic conditions that can predispose the development of MALS. The first condition is a higher origin of the celiac artery and the second condition is a lower positioning of the median arcuate ligament surrounding the abdominal aorta. Compression of the celiac artery usually occurs during expiration due to the upward movement of vascular structures caused by diminishing lung volumes in the thorax, which slightly shifts the thoracic and abdominal organs cephalically. Compression is also more likely when the median arcuate ligament is composed of thicker fibrous tissue or thin bands of tissue at or near the origin of the celiac artery [1].

As previously shown in this case presentation, MALS clinical presentation is vague and nonspecific. Dunbar syndrome’s presentation usually involves females having thin body habitus in the third to fifth decade of life. The main presentation includes postprandial epigastric pain, nausea, vomiting, diarrhea, and unexplained weight loss [1]. A study performed by Cusati et al. exposed the main symptoms presented, which included abdominal pain being the most common, predominantly postprandial abdominal weight. Then other symptoms presented were weight loss in half the patients studied, bloating, nausea, vomiting, and abdominal pain triggered by exercise in 8% of the patients [6]. In another study, performed by Park et al., a relation between chemoembolization of hepatic tumors and celiac stenosis was described, also in these patients, postprandial abdominal pain was the main symptom presented [7]. MALS is a diagnosis of exclusion and can mimic other abdominal disorders. Initial testing can be done using Duplex ultrasonography to screen for celiac artery compression; however, as with all ultrasonography screening, it is operator-dependent and requires an experienced operator in order to diagnose accurately and detect vascular changes during inspiration and expiration [8,9].

Computed tomography angiogram (CTA) is another useful tool to diagnose MALS since it allows for a three-dimensional visualization of the celiac artery and may better depict the compression of this structure. CTA reconstructs images with high resolution and may also show vascular changes associated with stenosis such as post-stenotic dilation. In contrast to ultrasonography, CTA uses ionizing radiation to produce images of internal organs, which might be a limiting factor in patients with renal dysfunction. Magnetic resonance angiography (MRA) is an alternative imaging modality for patients who are allergic to the intravenous contrast used in CTA. To be able to diagnose this condition by imaging, there must be a significant stenosis at the origin of the celiac trunk, greater than 50%, in a segment of at least 5 mm, with post-stenotic dilation and a deflection angle of the celiac trunk greater than 60° [10]. This criterion was met by our patient. Conventional angiography is currently the gold standard to show vascular flow changes due to dynamic compression of arteries as is the case during inspiration and expiration. Collateral flow development during expiration is a suggestive finding of celiac artery compression and must be carefully analyzed during evaluation with imaging. Collateral flow will usually be evidenced in the superior mesenteric artery (SMA) [3].

The management of MALS is based on the decompression of the celiac artery, allowing to establish an adequate blood flow and pain management. Treatment is based on the idea of treating neuropathic pain caused by celiac plexus neurolysis [11]. The definitive management is surgery. However, surgical options have changed in the last decades. Historically, open surgery used to be the gold standard treatment where a direct dissection and separation of the diaphragmatic crura from the celiac axis was performed [1]. Also, the neuropathic pain can be directly addressed by ablation or surgical removal of the celiac plexus ganglion. In the last decades, with the advancement of laparoscopic surgery, the first choice treatment in candidates is the laparoscopic release of the celiac artery. The advantages of this technique are a lower risk of complications associated with the surgery itself such as bleeding, increased surgical or anesthetic time, and smaller incisions. The main disadvantage presented with the laparoscopic approach is the incomplete release and potential risk of injury to the abdominal aorta [12]. Another important advancement was the emerging use of IR. Although IR has made great advances in medicine in recent years and represents one of the new fields of medicine with possibly the greatest impact, in the context of MALS, its role has not been as successful as monotherapy; this has been associated with the extrinsic compression of the celiac artery causing intimal hyperplasia that results in the luminal narrowing of the artery [2]. The use of PTA as an adjuvant therapy prior to surgery has shown benefits; also, there is an important emerging role of PTA in post-surgical recurrence or patients with residual symptoms after surgery with the addition of balloon expandable stents [13]. Nowadays, the trend is to perform a laparoscopic release with the use of an intraoperative duplex ultrasound [3].

## Conclusions

MALS is an infrequent condition and remains, therefore, a diagnosis of exclusion. Other abdominal conditions must be first explored in order to rule out other more frequent diseases. Diagnosis of MALS is centered on the visual depiction of the condition using different imaging modalities such as ultrasonography, tomography, resonance imaging, or angiography. Treatment is centered on laparoscopic approaches to release the compression of the celiac artery by the median arcuate ligament while minimizing complications that are more frequently associated with open abdominal approaches such as excess bleeding, increased postoperative pain, longer hospital length of stay, and worse cosmetic results. Although this condition might be infrequent, it must remain a differential diagnosis during evaluation in order to avoid unnecessary testing, ionizing radiation exposure, and excess economic and physical burden to the patient. This case is unique in its simultaneous presentation of a rare clinical entity and an anatomical variant. The implementation of a laparoscopic approach was crucial, as it facilitated the exposure of all vessels, enabling precise identification and thereby preventing vascular injuries. At the same time, it allowed the re-establishment of an adequate arterial flow to the abdominal organs.
